# Recurrent Biallelic p.L347P *PINK1* Variant in Polynesians with Parkinsonism and Isolated Dopa‐Responsive Dystonia

**DOI:** 10.1002/mdc3.13467

**Published:** 2022-07-02

**Authors:** Hugo Morales‐Briceno, Tien Lee Ong, Stephen R. Duma, Natalia Murray, Elizabeth M. Pepper, Ainhi Ha, Michel C. Tchan, Victor S.C. Fung

**Affiliations:** ^1^ Movement Disorders Unit, Department of Neurology Westmead Hospital Westmead New South Wales; ^2^ Sydney Medical School The University of Sydney Sydney New South Wales; ^3^ Department of Neurology John Hunter Hospital New Lambton New South Wales Australia; ^4^ Department of Genetic Medicine Westmead Hospital Westmead New South Wales Australia

**Keywords:** PINK1, dopa‐responsive dystonia, early‐onset Parkinson's disease, Pacific Islands, Polynesians

In studies of Caucasians with recessive, monogenic early‐onset Parkinson's disease (EOPD), mutations in *Parkin, PINK1 and DJ1* are most commonly identified.^1^ The prevalence of genotypes in the Asian Pacific population remains undetermined. Recently, eight Polynesian patients with PD harboring the homozygous p.L347P *PINK1* variant were reported, raising the possibility of a founder effect.^2^ We report four additional patients with this genotype, including isolated dopa‐responsive dystonia (DRD) in two.

The patients are unrelated but of Tongan, and one also of Samoan, descent. Patient 1 presented at 19 when he began to limp while playing rugby. Four years later, he developed progressive stiffness and involuntary posturing of the lower limbs with truncal flexion on walking. He reported marked diurnal variation as he was almost normal in the morning, had significant deterioration in the afternoon and was worst in the evening. At 25, he was diagnosed with lower limb dystonia and prescribed trihexyphenidyl 6 mg daily, with 75% improvement in his symptoms. Levodopa was added, and at a dose of 300 mg daily he had 90% improvement. At age 25 examination showed isolated leg and truncal dystonia (Video 1). Cerebrospinal fluid (CSF) analysis showed low biopterin of 9.9 nmol/L (25–45), borderline homovanillic acid of 0.09 μmol/L (0.09–0.37), normal neopterin 13.1 nmol/L (6–30) and 5‐HIAA at 0.07 μmol/L (0.06–0.19). Single gene analysis of *GCH1* found no pathogenic variants. Subsequently, a dystonia‐parkinsonism gene panel revealed homozygous p.L347P variants in *PINK1*. Patient 2 presented at age 40 with limping due to right lower limb stiffness. This gradually worsened with abnormal posturing of his lower limbs and slowed gait, particularly in the afternoon. Physical examination revealed isolated leg dystonia while walking. Levodopa 150 mg daily markedly reduced his symptoms leading to a suspicion of DRD. Monoamine CSF examination was not performed. He was also found to have homozygous p.L347P variants in *PINK1* when tested through a PD gene panel. Patients 3 and 4 presented with levodopa responsive EOPD at age 31 and 44 respectively. Detailed description of all four cases are shown in Data S1.

**Video 1:** Patient 1 was examined after 12 hours without levodopa (overnight withdrawal). There is no rest tremor, and finger taps were of normal amplitude and velocity. There were single interruptions on finger and foot tapping on the right side, however, no progressive decrement was seen. There was no facial hypomimia. During walking forwards, the left leg had a reduced knee flexion during the swing phase, with mild foot extension and eversion and toe extension. Also, he had flexion of the trunk while walking forwards, which was less prominent walking backwards.

Our observations strengthen the association between homozygous p.L347P *PINK1* mutations and EOPD in Polynesians, which also can present as isolated dopa‐responsive dystonia as shown in our cases. Lower limb dystonia is well‐recognized as occurring in EOPD in *PINK1* mutations, and was present in 5 of the 8 Polynesian patients with biallelic *PINK1* p.L347P variants recently reported by Patel et al.^2^ However, marked diurnal fluctuations, isolated dystonia and exquisite levodopa‐responsiveness mimicking Segawa disease have been rarely reported in PINK1.^4^ Worldwide, a total of 62 different *PINK1* pathogenic variants have been reported. The missense c.1040T > C (p. L347P) variant was initially reported in the Filipino population, and then in other patients from Taiwan, Guam and Malaysia.^3^, ^4^, ^5^, ^6^ Heterozygous p. L347P carrier status was identified in 3% of Filipino controls in one study, whereas this variant was not detected in the European population.^7^ Furthermore, Patel SG et al. estimated a carrier prevalence of 1.5% from 273 Maori and Pacific healthy controls.^2^ Our observations further support the likelihood of a founder effect in Polynesian patients with EOPD, and suggest it should also be considered in the differential diagnosis of isolated, dopa‐responsive dystonia in Pacific islanders.

## Author Roles

1) Research project: A. Conception, B. Organization, C. Execution; 2) Statistical Analysis: A. Design, B. Execution, C. Review and Critique; 3) Manuscript: A. Writing of the first draft, B. Review and Critique.

HM: 1A, B, C, 3A, 3B;

TLO: 1B, C, 3A, 3B;

SD: 1B, C, 3A, 3B;

NM: 1B, C, 3A, 3B;

EP: 1B, C, 3B;

AH: 1A, B, C, 3B;

MT: 1A, B, C, 3B;

VF: 1A, B, C, 3B.

## Disclosures

**Ethical Compliance Statement:** The authors confirm that the approval of an institutional review board was not required for this work. Informed consent for publication of video/images was obtained from all patients described here. We confirm that we have read the Journal's position on issues involved in ethical publication and affirm that this work is consistent with those guidelines.

**Funding Sources and Conflicts of Interest:** The authors report no specific grants for this research financial disclosure or conflicts of interest.

**Financial Disclosures for the previous 12 months:** Dr Fung receives a salary from NSW Health, has received unrestricted research grants from Michael J Fox Foundation, Abbvie and Merz, is on Advisory Boards and/or has received travel grants from Abbvie, Allergan, Ipsen, Merz, Praxis, Seqirus, Stada, Teva and UCB, and receives royalties from Health Press Ltd. Dr Morales receives salary from NSW Health. Dr Duma receives salary from NSW Health. Dr Murray receives salary from NSW Health. Dr Pepper receives salary from NSW Health. Dr Ha receives salary from NSW Health. Dr Tchan receives salary from NSW Health. Dr Ong reports no financial disclosures.

## Supporting information


**Data S1.** Clinical characteristics of four patients with the p.L347P PINK1 genotype.
