# Supplementary material for: Recurrent Biallelic p.L347P PINK1 Variant in Polynesians with Parkinsonism and Isolated Dopa‐Responsive Dystonia
Source: Mov Disord Clin Pract. 2022 Jul 2;9(5):696–7. doi: 10.1002/mdc3.13467 (PMC9274338; doi:10.1002/mdc3.13467)
Supplement: Supplementary file 1 — Data S1. Clinical characteristics of four patients with the p.L347P PINK1 genotype. [file MDC3-9-696-s001.docx]

**Supplementary material**

**Clinical characteristics of four patients with the p.L347P PINK1 genotype**

Patient 1 had normal early development, presenting at 19 when he began to limp while playing rugby. There was no family history of Parkinsonism. Four years later, he developed progressive stiffness and involuntary posturing of the lower limbs with truncal flexion on walking. He reported marked diurnal variation as he was almost normal in the morning, had significant deterioration in the afternoon and was worst in the evening. At 25, he was diagnosed with lower limb dystonia and prescribed trihexyphenidyl 6 mg daily, with 75% improvement in his symptoms. Levodopa was added, and at a dose of 300 mg daily he had 90% improvement. The video shows him at age 25 years, off levodopa treatment. There is reduced left knee flexion with left toe extension and mild foot eversion during the swing phase. There was also a slight dystonic posturing of the right toes while walking forwards. Gait was normal when running or walking backwards. There was no rigidity, bradykinesia or tremor, and the neurological examination was otherwise normal. Brain MRI was normal. Cerebrospinal fluid (CSF) analysis showed low biopterin of 9.9nmol/L (25-45), borderline homovanillic acid of 0.09µmol/L (0.09-0.37), normal neopterin 13.1nmol/L (6-30) and 5-HIAA at 0.07µmol/L (0.06-0.19). Targeted GCH1 genetic analysis found no pathogenic variants. Subsequently, a dystonia-parkinsonism gene panel revealed homozygous p.L347P mutations in PINK1. Ten years after the presentation with dystonia, he developed mild Parkinsonism with motor fluctuations.

Patient 2 presented at age 40 with limping due to right lower limb stiffness. This gradually worsened with abnormal posturing of his lower limbs and slowed gait, particularly in the afternoon. Physical examination revealed left foot external rotation with forefoot strike and reduced bilateral arm swinging while walking. However, he was able to run normally. There was no rigidity, bradykinesia or tremor, and the neurological examination was otherwise normal. Levodopa 150 mg daily markedly reduced his symptoms leading to a suspicion of DRD. He was also found to have homozygous p.L347P mutations in PINK1. Six months after the initiation of levodopa, he developed motor fluctuations. There was no family history of Parkinsonism.

Patient 3 is of Tongan decent but was born in Australia. He has no parental consanguinity or family history of neurological disorder. In his mid-20s, he developed a sensation of right knee numbness and several months later, he noticed intermittent right leg tremor at rest. In the subsequent few years, he noticed the development of truncal stiffness, and was dragging his right leg while walking. He was diagnosed with EOPD at the age of 31 and was prescribed with levodopa, building up to a dose of 1000 mg daily and later added on rasagiline 1mg daily. The response to levodopa was noticeable, and two years after treatment, he developed motor fluctuations and dyskinesias. Clinical examination at the age of 34 showed there was no upper limb rest or postural tremor (Video 1). Finger and toe tapping were impaired on the right. There was right arm rigidity only with co activation of the left arm. The remainder of oculomotor, sensory, cerebellar and motor examination were normal. Brian MRI was normal. His genetic test revealed homozygous p.L347P PINK1 mutation.

Patient 4 was born in Samoa and migrated to Australia. His parents are unaffected and he has four children that are healthy. He presented at the age of 44 with dragging of the left leg. This was associated with progressive slowness and stiffness resulting in a gradual impairment of mobility and fine motor function. He developed a bilateral upper limb tremor at age 50, which was most noticeable during action or during sustained postures. He was started on levodopa/carbidopa 100/25 mg three times a day at that age with excellent symptom control to the point he could mobilise again with any walking aid. Examination at age 53 he had mild dyskinesia in the lower limbs, with moderate bradykinesia in upper limbs and lower libs, which was more pronounced in the right. He had mild limb rigidity and there was no rest tremor. He had a mild postural and action tremor. Pull test was normal. He reports anxiety and depression, but find interest and joy in looking after his family and remains motivated. No hallucinations, no incontinence but mild urinary urgency. No falls no freezing of gait. The remainder of oculomotor, sensory, cerebellar and motor examination were normal. Brian MRI was normal. His genetic test revealed homozygous p.L347P PINK1 mutation.
